# P-222. Anal Cancer Screening in People with HIV with Limited Access to High Resolution Anoscopy

**DOI:** 10.1093/ofid/ofaf695.444

**Published:** 2026-01-11

**Authors:** Arati B Pandya, Aarjav P Pandya, Sarah H Yates, Brooke A Rabe, Krishna C Babaria, Jesse E Ritter, Catherine Anania, Janardan S Sivapalan, Lori E Fantry

**Affiliations:** University of Arizona College of Medicine - Tucson, Tucson, Arizona; University of Arizona College of Medicine - Tucson, Tucson, Arizona; University of Arizona College of Medicine - Tucson, Tucson, Arizona; University of Arizona, Tucson, Arizona; University of Arizona College of Medicine - Tucson, Tucson, Arizona; University of Arizona College of Medicine - Tucson, Tucson, Arizona; University of Arizona Department of Medicine/ Banner University Medical Center- Tucson, Tucson, Arizona; University of Arizona College of Medicine - Tucson, Tucson, Arizona; University of Arizona College of Medicine - Tucson, Tucson, Arizona

## Abstract

**Background:**

Anal cancer incidence is increasing in the United States with an estimated 9,540 new cases in 2024. People with HIV (PWH) face the highest risk. The ANCHOR trial demonstrated that treating high-grade squamous intraepithelial lesions (HGSIL) in PWH significantly reduces anal cancer risk compared to controls. Screening with anal cytology (Pap smear) and high-resolution anoscopy (HRA), when indicated, is recommended for PWH but remains inadequate globally, including in the US. This study evaluates anal cancer screening in a region where HRA access requires at least one-hour travel.

**Figure 1.**
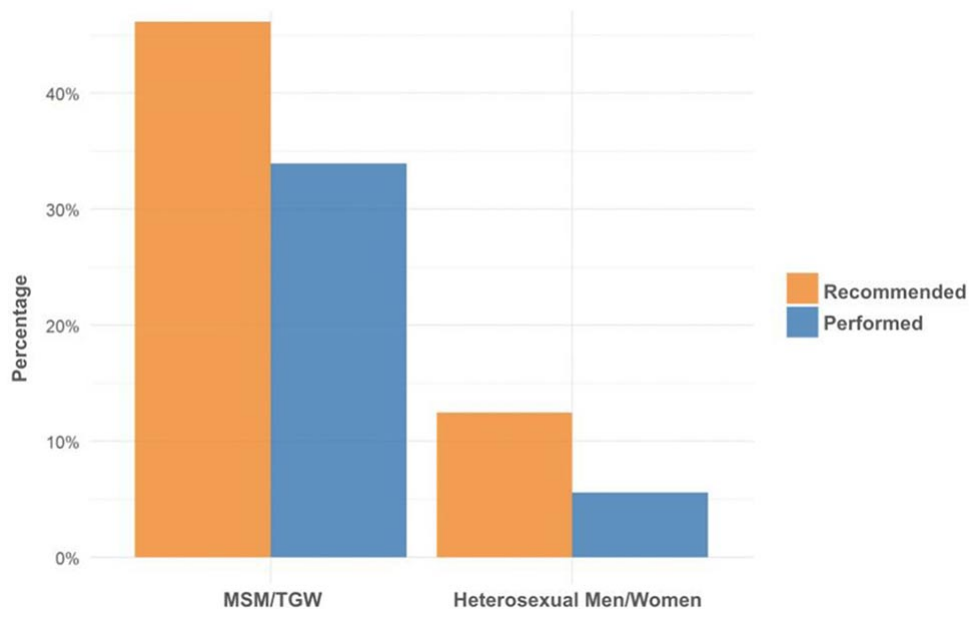
Percentages of Anal Pap Smears Recommended and Performed Sexual orientation influences whether an anal Pap smear is recommended and performed.

**Figure 2.**
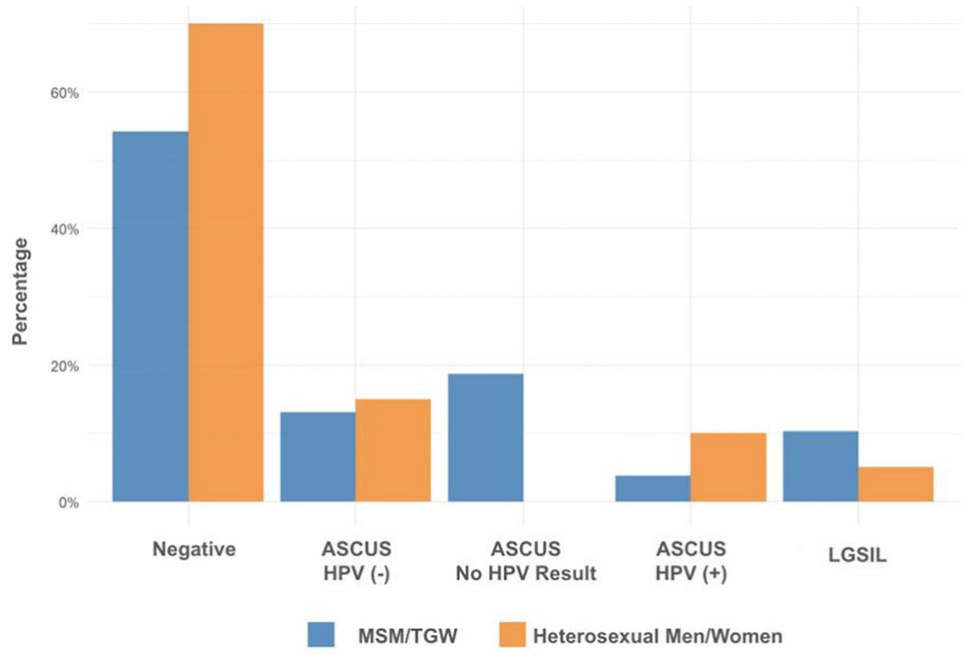
Anal Pap Smear Results Most individuals had negative anal Pap smear results, but this difference did not reach statistical significance (p value = 0.171).

**Methods:**

This retrospective study included two Ryan White-funded HIV clinics in Tucson, Arizona. Eligible participants, based on International Anal Neoplasia Society guidelines, were men who have sex with men (MSM) and transgender women (TGW) aged 35–75, and heterosexual men and women aged 45–75, with at least one clinical encounter between March 1, 2022, and January 14, 2025, and an anal Pap smear performed. Charts from five of twelve providers who reported not performing anal Paps were excluded. Proportions were compared using chi-square or Fisher’s exact tests, as appropriate.

**Figure 3.**
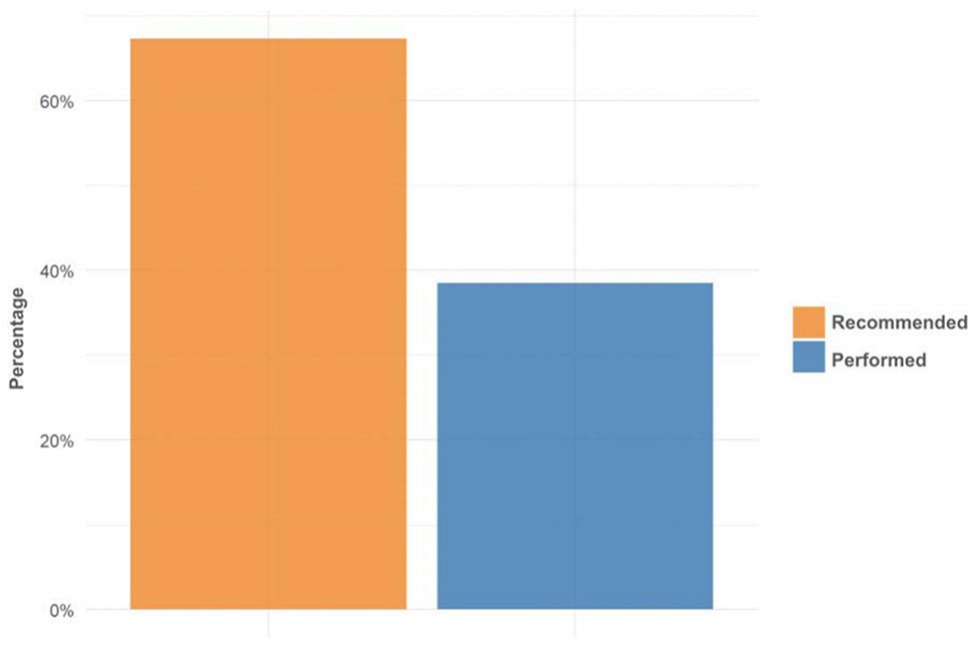
Percentages of HRA Recommended and Performed 38.5% of individuals who were recommended HRA had it performed.

**Figure 4.**
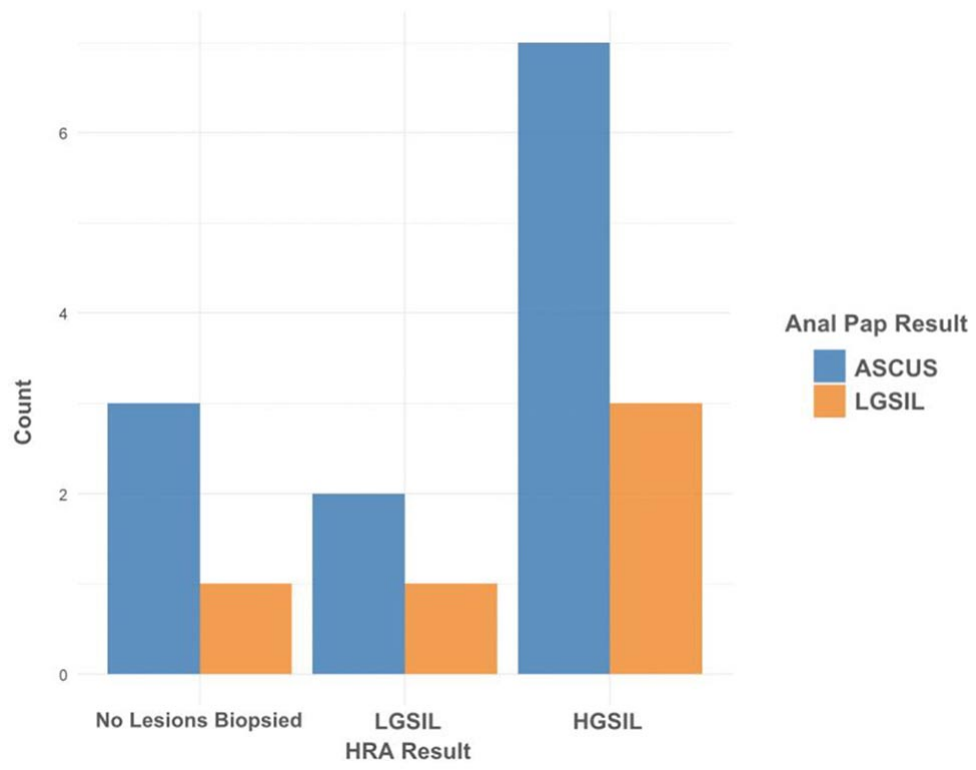
HRA vs Anal Pap Smear Results Anal Pap results did not predict HRA results.

**Results:**

Of 377 eligible individuals, 266 (70.6%) were MSM, 58 (15.4%) heterosexual men, 48 (12.7%) women, 4 (1.1%) TGW, and 1 (0.3%) transgender man. Anal Paps were recommended for 174/270 (64.4%) MSM and TGW and performed in 128 (47.4%) compared to lower rates in other groups (p value < 0.001) (Fig. 1). Most results were negative; atypical squamous cells of undetermined significance (ASCUS), with or without HPV, was the second most common finding (Fig. 2). No HGSIL was detected. Regardless of sexual orientation, 20/35 individuals underwent HRA when recommended (Fig. 3). Anal Pap results showed poor correlation with HRA findings (Fig. 4).

**Conclusion:**

Anal cancer screening is challenging particularly when patients perceive their risk for anal cancer is low. Individuals at higher risk for anal cancer (MSM and TGW) were more likely to be recommended and pursue screening than heterosexual individuals. Ryan White funding, as we used, can help pay for HRA and transportation to it. However, local HRA, planned for Tucson in 2025, is needed to improve screening uptake and outcomes in all groups.

**Disclosures:**

All Authors: No reported disclosures

